# HIV-1 CA Inhibitors Are Antagonized by Inositol Phosphate Stabilization of the Viral Capsid in Cells

**DOI:** 10.1128/JVI.01445-21

**Published:** 2021-11-23

**Authors:** Gregory A. Sowd, Jiong Shi, Christopher Aiken

**Affiliations:** a Department of Pathology, Microbiology, and Immunology, Vanderbilt University Medical Center, Nashville, Tennessee, USA; Ulm University Medical Center

**Keywords:** HIV, IP6, capsid, CA, endogenous reverse transcription, GS-6207, lenacapavir, PF74, IPMK, IPPK

## Abstract

The HIV-1 capsid, composed of the CA protein, is the target of the novel antiretroviral drug lenacapavir (LCV). CA inhibitors block host factor binding and alter capsid stability to prevent nuclear entry and reverse transcription (RTN), respectively. Capsid stability is mediated *in vitro* by binding to the host cell metabolite inositol hexakisphosphate (IP6). IP6 depletion in target cells has little effect on HIV-1 infection. We hypothesized that capsid-altering concentrations of CA inhibitors might reveal an effect of IP6 depletion on HIV-1 infection in target cells. To test this, we studied the effects of IP6 depletion on inhibition of infection by the CA inhibitors PF74 and LCV. At low doses of either compound that affect HIV-1 nuclear entry, no effect of IP6 depletion on antiviral activity was observed. Increased antiviral activity was observed in IP6-depleted cells at inhibitor concentrations that affect capsid stability, correlating with increased RTN inhibition. Assays of uncoating and endogenous RTN of purified cores *in vitro* provided additional support. Our results show that inositol phosphates stabilize the HIV-1 capsid in target cells, thereby dampening the antiviral effects of capsid-targeting antiviral compounds. We propose that targeting of the IP6-binding site in conjunction with CA inhibitors will lead to robust antiretroviral therapy (ART).

**IMPORTANCE** HIV-1 infection and subsequent depletion of CD4^+^ T cells result in AIDS. Antiretroviral therapy treatment of infected individuals prevents progression to AIDS. The HIV-1 capsid has recently become an ART target. Capsid inhibitors block HIV-1 infection at multiple steps, offering advantages over current ART. The cellular metabolite inositol hexakisphosphate (IP6) binds the HIV-1 capsid, stabilizing it *in vitro*. However, the function of this interaction in target cells is unclear. Our results imply that IP6 stabilizes the incoming HIV-1 capsid in cells, thus limiting the antiviral efficiency of capsid-destabilizing antivirals. We present a model of capsid inhibitor function and propose that targeting of the IP6-binding site in conjunction with capsid inhibitors currently in development will lead to more robust ART.

## INTRODUCTION

The mature conical human immunodeficiency virus 1 (HIV-1) capsid is composed of the CA protein. In addition to serving as a shell that encases and protects the viral genomic RNA (1), the capsid serves as a docking platform for interactions with host proteins that promote intracytoplasmic transport, nuclear entry, and nuclear trafficking/integration site targeting (1–5). A specific interface in the capsid lattice is targeted by the antiretroviral compounds PF74 and lenacapavir (LCV) (formerly known as GS-6207) (6–8). Consistent with the CA binding site, LCV and PF74 inhibit multiple aspects of HIV-1 infection. At low concentrations of the inhibitors, nuclear entry is blocked (6, 7). At high concentrations of LCV or PF74, HIV-1 capsid stability is affected, leading to impaired reverse transcription (RTN) (7, 9). These characteristics of CA inhibitors along with the relative intolerance of CA to mutation (10) make CA an attractive drug target.

Changes in HIV-1 capsid stability directly influence the outcome of infection. Hyperstable capsids are proficient for RTN, but infection is blocked at subsequent steps (11). Conversely, HIV-1 mutants with unstable capsids typically fail to reverse transcribe viral RNA, resulting in abortive infection (11). Capsid stability is also influenced by host factor binding. The host metabolite inositol hexakisphosphate (IP6) binds to arginine 18 and lysine 25 in CA, residues proposed to facilitate nucleotide import and distinct from those that bind to LCV/PF74 (3, 7, 12–15). IP6 association with CA stabilizes the capsid *in vitro*, thus permitting the synthesis of full-length reverse transcripts in endogenous RTN (ERT) reactions using purified cores and permeabilized virions (16, 17). However, in cells, depletion of IP6 and the related metabolite inositol (1, 3, 4, 5, 6) pentakisphosphate (IP5) via knockout (KO) of the genes encoding inositol polyphosphate multikinase (IPMK) or inositol-pentakisphosphate 2-kinase (IPPK), the cellular kinases required for IP5 and/or IP6 biogenesis, has a relatively minor effect on target cell susceptibility to HIV-1 infection (18, 19). CA and Gag substitutions that perturb IP6 binding also perturb mature virion assembly; however, it is difficult to separate the effects of the mutations themselves on capsid stability and structure from those on IP6 binding (11, 14, 20). Thus, the consequences of CA binding to IP6 in the target cell for HIV-1 infection remain unclear.

The PF74/LCV binding site on CA is shared with several host proteins, and depletion of these cellular proteins has been demonstrated to affect drug potency or antiviral activity (2, 3, 7, 21, 22). However, little is understood about how other host factors that bind CA surfaces distinct from the LCV binding site on CA influence LCV/PF74 antiviral activity. Further, we sought to examine the role of inositol phosphates in HIV-1 infection in T cells. To this end, IP6- or IP5/IP6-depleted T cell lines (18) were used as target cells for HIV-1 infection in the presence or absence of CA inhibitors. A cryptic, inositol phosphate-dependent defect in HIV-1 RTN was revealed only in IP6- and IP5/IP6-depleted target cells dosed with PF74 or LCV. Further, by performing *in vitro* assays with purified HIV-1 cores, we determined that PF74 and LCV destabilize the HIV-1 core via distinct mechanisms that can be antagonized by increasing the amount of IP6 in the reaction. Thus, our cellular and *in vitro* studies indicate that the association of IP6 with CA stabilizes the HIV-1 capsid in cells during RTN. Our data present a model wherein IP6 stabilizes the HIV-1 capsid, thereby reducing the effects of capsid-destabilizing CA inhibitors.

## RESULTS

### IP6 decreases PF74 antiviral activity by enhancing RTN.

**FIG 1:**
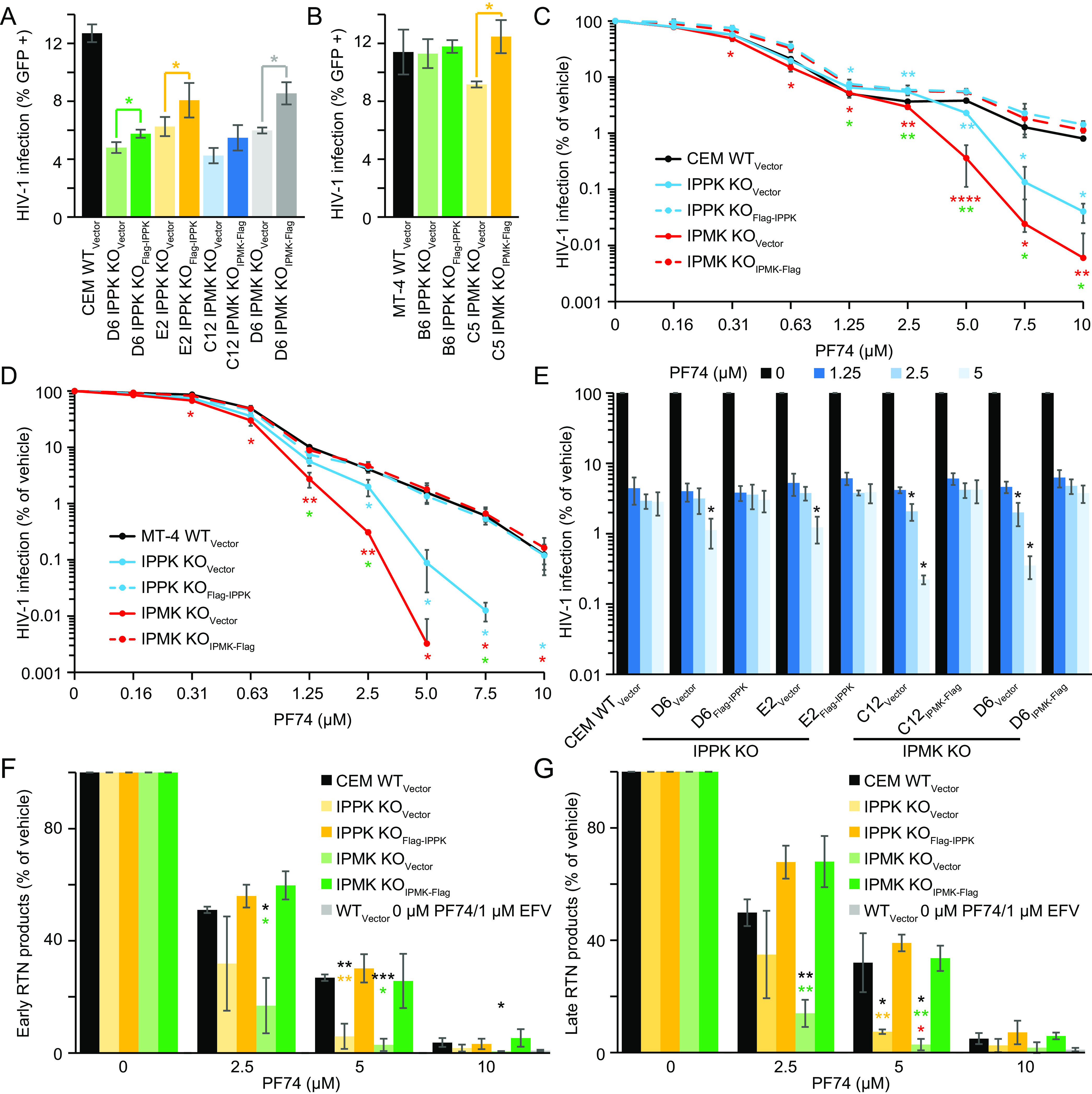
Depletion of IP5 and/or IP6 increases HIV-1 susceptibility to PF74. (A and B) Efficiency of HIV-1-GFP_VSVg_ infection by flow cytometry when the indicated CEM (A) or MT-4 (B) cell lines are used as targets for infection. (C and D) PF74 dose-response curves for HIV-1-GFP_VSVg_-infected CEM- (C) or MT-4-derived (D) cell lines as measured by flow cytometry. No HIV-1-infected cells were detected when IPMK KO MT-4 cells were infected in the presence of 7.5 and 10 μM PF74. For IPPK KO MT-4 cells, no infected cells were detected in the presence of 10 μM PF74. Cyan and red asterisks represent significance when comparing HIV-1 infection of IPPK KO_Vector_ and IPMK KO_Vector_ cells, respectively, to that of the corresponding complemented cell line. Green asterisks denote significantly different HIV-1 infection of IPPK KO_Vector_ cells to that of IPMK KO_Vector_ cells. (E) Target cell HIV-1-GFP_VSVg_ infection efficiency of the indicated CEM cell lines as measured by flow cytometry. Asterisks signify significance of the designated KO_Vector_ cell line compared to the corresponding complemented cell line. (F and G) Early (FST) (F) or late (FLM) (G) RTN products at 8 h following infection with HIV-1_VSVg_ as measured by quantitative PCR (qPCR). The nonnucleoside reverse transcriptase inhibitor Efavirenz (EFV) was used to identify the background of the assay. Black asterisks indicate significantly different susceptibility to HIV-1 infection between WT_Vector_ cells and the designated KO_Vector_ cell line. Yellow and green asterisks signify significance between infection of the IPPK KO_Vector_ cells or IPMK KO_Vector_ cells, respectively, relative to the corresponding complemented cell line. Each panel displays the average of 3 independent experiments with error bars representing standard deviation. Significance levels are as follows: *, *P* < 0.05; **, *P* < 0.01; ***, *P* < 0.001; ****, *P* < 0.0001.

Whereas IPPK KO depletes most, if not all, of the cellular IP6, IPMK KO depletes both IP5 and IP6 (18, 19, 23). We previously noted that KO of either kinase in CEM CD4^+^ T cells lowered target cell susceptibility to HIV-1 infection by ∼2-fold and in MT-4 CD4^+^ T cells IP6 depletion had little to no effect on target cell HIV-1 infection (18). However, it was unclear if complementation would influence this outcome. To test this, the wild-type vector (WT_Vector_), KO_Vector_, and complemented CEM and MT-4 cells were infected with vesicular stomatitis virus G protein (VSVg)-pseudotyped HIV-1 expressing green fluorescent protein (GFP) from the *nef* position (HIV-GFP_VSVg_), and the extent of infection was scored by flow cytometry. In CEM cells, KO of either kinase decreased HIV-1 infection by greater than 2-fold compared to WT_Vector_ cells (Fig. 1A). Regardless of CEM clone tested, retroviral complementation increased target cell susceptibility to HIV-1 infection (Fig. 1A, Flag-IPPK and IPMK-Flag cell lines), though not to the level observed in the original CEM cell line. In the MT-4 cell panel (18), IP6 depletion via IPPK KO had no effect on target cell susceptibly to HIV-1 infection nor did IPPK complementation (Fig. 1B). Relative to MT-4 WT_Vector_ cells, IPMK KO subtly decreased HIV-1 infection; however, upon IPMK complementation, a larger fraction of target cells was infected (Fig. 1B). Our results suggest that in target cells, IP5 and IP6 have a relatively minor effect on HIV-1 infection.

Since a minor effect of IP6 depletion on susceptibility to HIV-1 infection was observed, we hypothesized that additional capsid destabilization might be required to reveal an effect. The CA inhibitor PF74 increasingly destabilizes the HIV-1 capsid in a dose-dependent manner, requiring PF74 concentrations greater than 1.25 μM to destabilize the capsid *in vitro* (9). We infected the CEM or MT-4 KO cell panels with HIV-GFP_VSVg_ over a wide range of PF74 concentrations and quantified the extent of infection by flow cytometry. As expected, HIV-1 exhibited a biphasic dose-response curve in the presence of PF74 in WT_Vector_ and both complemented cell lines (Fig. 1C and D). IPMK or IPPK KO had a minor effect on HIV-1 infection when cells were dosed with PF74 at concentrations of 1.25 μM or lower (Fig. 1C and D; Table 1). At these concentrations, PF74 primarily inhibits nuclear entry and integration (6, 24). By contrast, at PF74 concentrations of 2.5 μM or greater, HIV-1 infection of the KO_Vector_ cell lines was progressively reduced to a greater extent relative to WT_Vector_ and the respective complemented cell lines (Fig. 1C and D). HIV-1 infection was inhibited by greater than 20-fold in IPPK KO_Vector_ cells dosed with 10 μM (CEM) or 5 μM (MT-4) PF74. Furthermore, 10 μM (CEM) or 5 μM (MT-4) PF74 inhibited HIV-1 infection by greater than 100-fold in IPMK KO_Vector_ cells relative to complemented cells. Regardless of T cell line tested, the enhanced PF74 antiviral activity resulting from IP5 and/or IP6 depletion was associated with a shorter plateau phase and a corresponding increase in the terminal slope (Hill coefficient, m′) of the inhibition curve (Fig. 1C and D; and Table 1, m′). At these concentrations, HIV-1 infection was more robustly inhibited in IPMK KO_Vector_ cell lines versus IPPK KO_Vector_ cell lines (Fig. 1C and D). The enhanced PF74 antiviral activity in cells depleted of IP5 and IP6 was observed regardless of cell line tested or clone used (Fig. 1E). Thus, we conclude that IP6 reduces the antiviral efficiency of PF74 at concentrations of the compound that were previously shown to destabilize the HIV-1 capsid.

**TABLE 1:** Hill equation parameters and fit for IPMK or IPPK KO effect on PF74 inhibition of HIV-1 infection in T cells

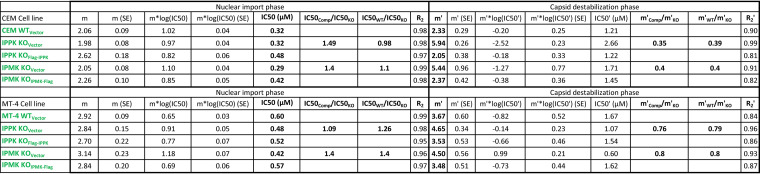

High concentrations of PF74 inhibit HIV-1 RTN during target cell infection (3, 9, 21). We therefore tested if the increase in PF74 antiviral activity in IP6-depleted cells resulted from a more potent inhibition of RTN by PF74. To this end, the CEM cell lines were dosed with PF74, infected with HIV-1, and RTN was assessed at 8 h by quantitative PCR (qPCR). In each CEM cell line, 2.5 μM PF74 blocked RTN, albeit to differing extents (Fig. 1F and G). WT_Vector_ and complemented cell lines contained similar amounts of HIV-1 cDNA independent of locus tested (Fig. 1F and G). IP6 depletion had a small effect on RTN at 2.5 μM PF74; however, RTN was decreased compared to controls at 5 μM PF74 (Fig. 1F and G; Table 2). At all tested PF74 concentrations IPMK KO decreased HIV-1 first strand transfer (FST) products to a greater extent than those observed in WT_vector_, IPMK KO_IPMK-Flag_, and IPPK KO_Vector_ cell lines (Fig. 1F and Table 2). PF74 affected full length minus strand (FLM) synthesis nearly identically to FST synthesis regardless of cell line tested (Fig. 1G). Our results indicate that endogenous levels of IP6 antagonize PF74 action during HIV-1 infection by counteracting inhibition of RTN by the antiviral compound.

**TABLE 2:** Hill equation parameters and fit for the effect of IP5/IP6 depletion on inhibition of HIV-1 RTN by PF74 in CEM cells

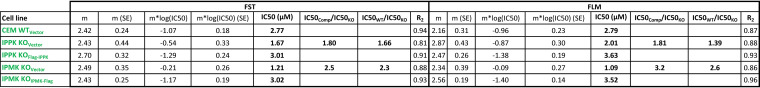

### LCV antiviral efficiency is affected by IP6 depletion.

**FIG 2:**
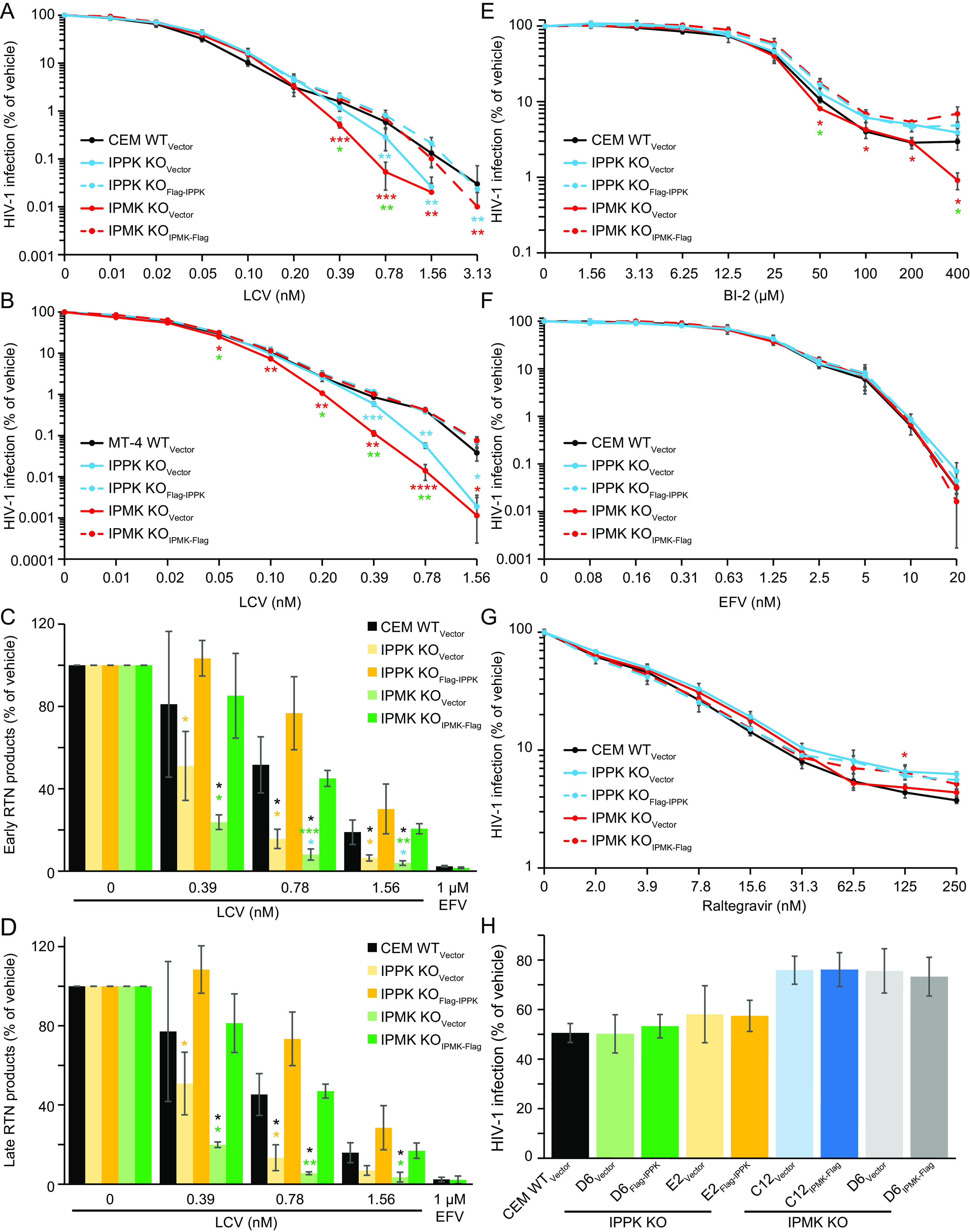
LCV more potently inhibits HIV-1 RTN upon IPMK or IPPK KO. (A and B) Target cell HIV-1-GFP_VSVg_ infection in the presence of LCV measured by flow cytometry. (A) Zero infected IPMK KO cells were detected when cells were infected in the presence of 3.1 nM LCV. (C and D) Quantification of early (FST) (C) or late (FLM) (D) RTN products. Black asterisks signify significance of the denoted bar from WT_Vector_ cells. Yellow and green asterisks above a bar indicate significance of that bar from the corresponding complemented cell line. (E to H) Target cell HIV-1-GFP_VSVg_ infection of the indicated cell lines when dosed with BI-2 (E), EFV (F), Raltegravir (G), or cyclosporine A (H). An average of 3 independent experiments is shown with error bars for standard deviation. Significance levels are as follows: *, *P* < 0.05; **, *P* < 0.01; ***, *P* < 0.001.

Although LCV shares a binding site with PF74, LCV appears to have a distinct effect on the HIV-1 capsid, stabilizing rather than destabilizing it (7). Thus, the effect of inositol phosphate depletion on HIV-1 in the presence of LCV might differ when compared to those observed using PF74. To test this, the CEM and MT-4 cell panels were dosed with increasing amounts of LCV and infected with HIV-1. In both cell lines, WT_Vector_ and both complemented cell lines displayed a biphasic inhibition curve with an inflection point occurring between 0.2 and 0.8 nM LCV (Fig. 2A and B). In IPPK and IPMK KO_Vector_ cell lines, we observed minimal changes in LCV sensitivity at LCV concentrations of less than 0.1 nM, which were previously associated with inhibition of HIV-1 nuclear entry (7). At LCV concentrations of 0.78 nM or higher, a significant increase in antiviral activity was observed in IPPK KO_Vector_ cells relative to WT_Vector_ and IPPK KO_Flag-IPPK_ cell lines (Fig. 2A and B). Further, IPMK KO resulted in enhanced LCV inhibition of HIV-1 infection in a dose-dependent manner (Fig. 2A and B), suggesting that IP5 and IP6 counter the antiviral activity of LCV. Independent of cell line tested and relative to complemented controls, IPMK KO and IPPK KO reduced HIV-1 infection by greater than 10-fold upon dosing cells with 0.78 or 1.56 nM LCV, respectively. In both IP6- and IP5/IP6-depleted cell lines, the slope of inhibition of HIV-1 infection by LCV became steeper at high concentrations with little to no effect on the 50% inhibitory concentration (IC_50_) (Table 3, m′). These data further suggest that IP6 has a function during the early phase of HIV-1 infection.

**TABLE 3:** Hill equation parameters and fit for the effect of IP5/IP6 depletion in T cells on LCV inhibition of HIV-1 infection

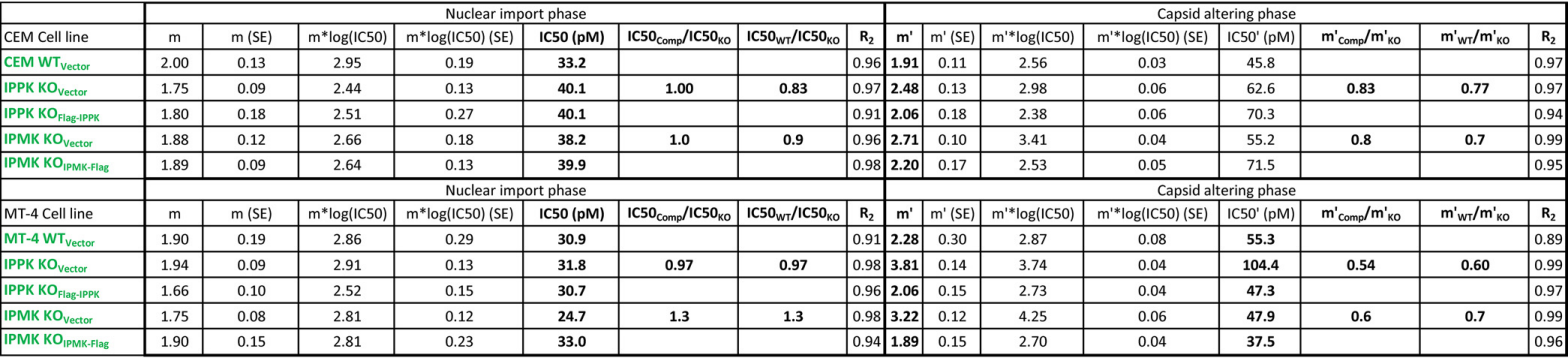

To gain more insight into what stage of HIV-1 infection was differentially affected upon LCV dosing of inositol phosphate-depleted cells, RTN in cells was quantified by qPCR. In WT_Vector_ and complemented cell lines, early RTN was inhibited by LCV concentrations of 0.78 nM or higher (Fig. 2C and D). However, upon IP6 depletion by IPPK or IPMK KO, the drug inhibited RTN at concentrations as low as 0.39 nM (Fig. 2C and D; Table 4). Similar to PF74, IPMK KO significantly inhibited HIV-1 RTN to a greater extent than IPPK KO (Fig. 2C and D). The IC_50_ of LCV for inhibiting HIV-1 RTN was altered by >2- and >4-fold upon IPPK KO and IPMK KO, respectively, contributing to the altered slope in the dose-response curve (Table 3, m′; Table 4, IC_50_). These results suggest that cellular IP6 decreases LCV efficiency by promoting RTN in cells.

**TABLE 4:** Hill equation parameters and fit for the effect of IP5/IP6 depletion on inhibition of HIV-1 RTN by LCV in CEM cells

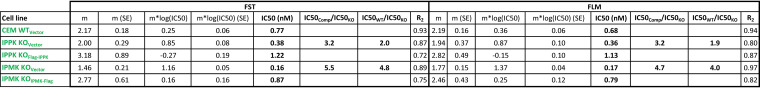

We next assessed the specificity of the effects of inositol phosphate depletion on inhibitors of early steps of HIV-1 infection. To this end, we tested whether inhibition of nuclear entry, reverse transcriptase, or integrase by BI-2 (25), efavirenz (EFV), or raltegravir, respectively, is affected by IP5 and/or IP6 depletion. KO of either kinase had little to no effect on the IC_50_ and slope of inhibition of any of these compounds (Fig. 2E to G; Table 5). A modest increase in the inhibition of HIV-1 infection by the CA-targeting compound BI-2 could be observed in IPMK KO cells at concentrations of >100 μM; however, at this concentration, the specificity of the drug becomes difficult to assess owing to cytotoxicity (Fig. 2E). Further, blocking cyclophilin A binding to CA with cyclosporine A had a similar effect on HIV-1 in all clones tested (Fig. 2H). Therefore, we conclude that the potentiation of PF74 and LCV antiviral activity during RTN inhibition in IP6-depleted cell lines results directly from alterations to the HIV-1 capsid during RTN and not some other aspect of infection.

**TABLE 5:** Hill equation parameters and fit for BI-2, EFV, or raltegravir inhibition of HIV-1 infection in CEM cells

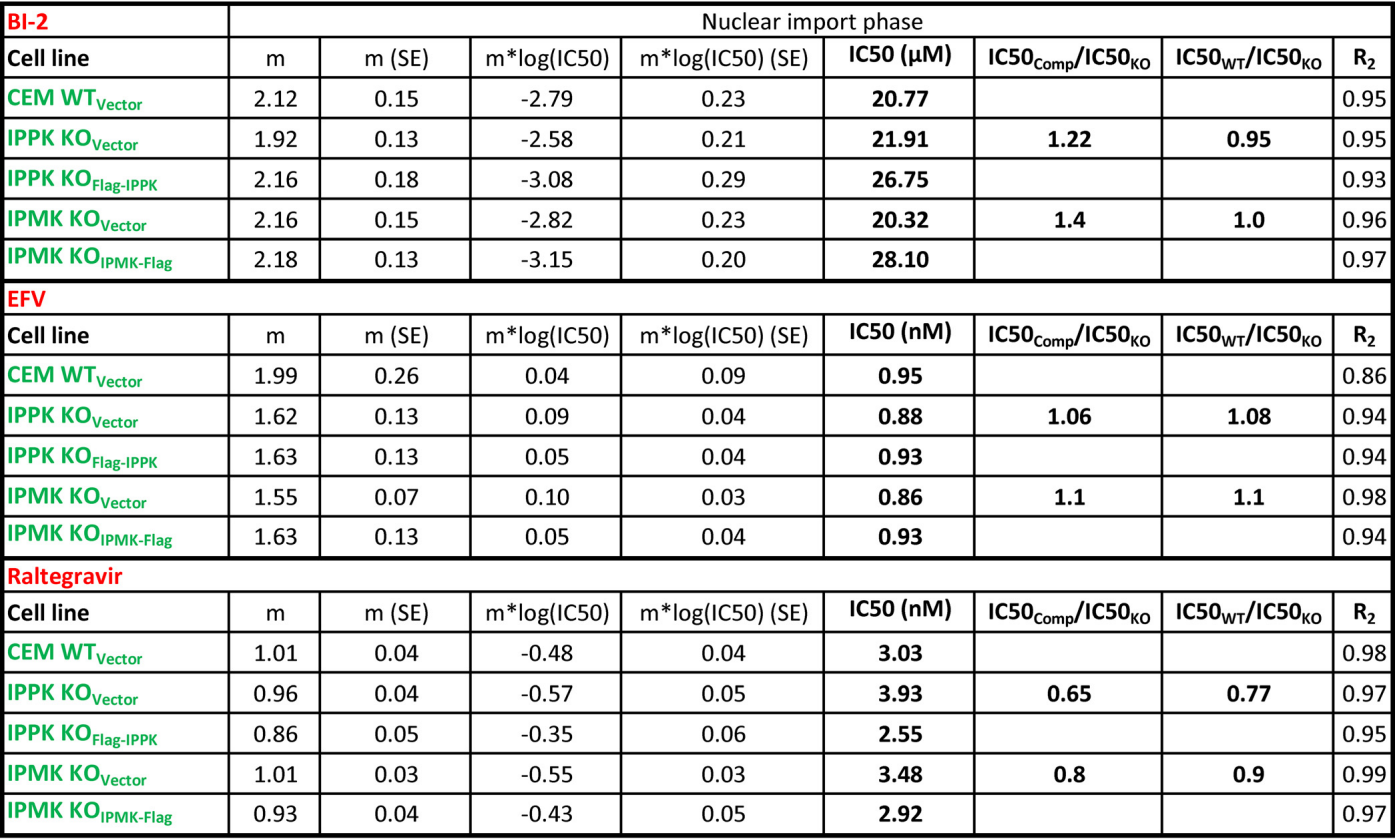

### IP6 promotes infection of LCV-resistant HIV-1 mutants.

**FIG 3:**
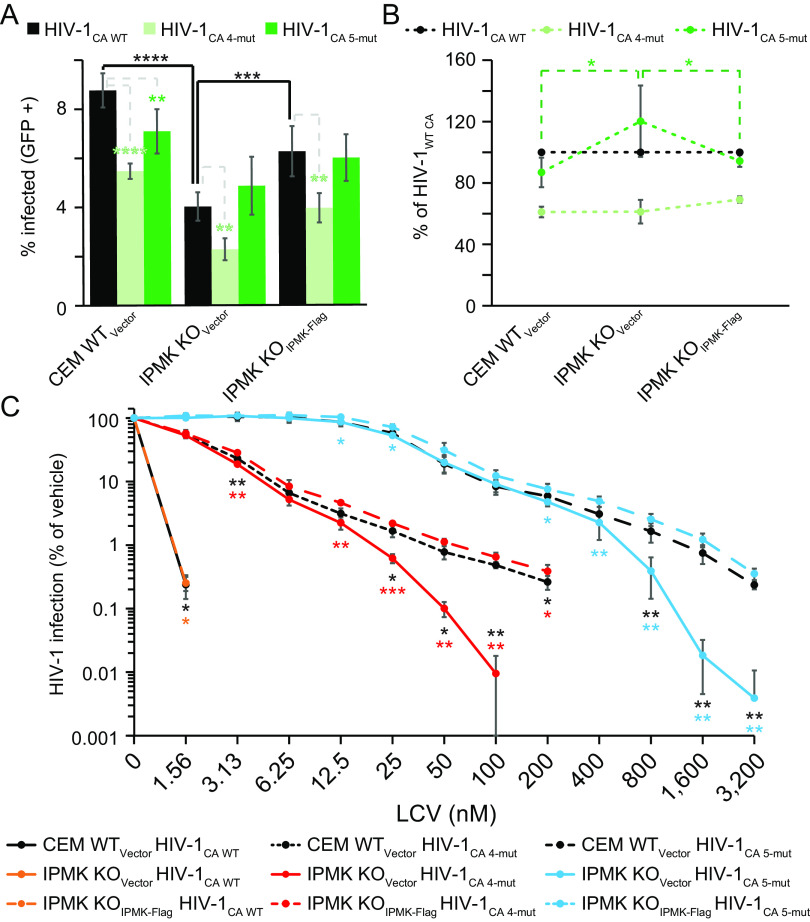
IP5 and IP6 depletion can partially sensitize PF74-resistant HIV-1 mutants to LCV. (A and B) Effect of CA 4-mut and CA 5-mut on HIV-1-GFP_VSVg_ infection of the indicated target cells. (C) LCV dose-response curves for HIV-1 CA 4-mut and CA 5-mut. Zero HIV-1_CA WT_-infected IPMK KO_Vector_ CEM cells were detected in cultures containing 1.56 nM LCV. Black asterisks denote significance between HIV-1-infected- WT_Vector_ cells and IPMK KO_Vector_ cells. Differences between HIV-1 infection of IPMK-Flag complemented cells and IPMK KO_Vector_ cells is indicated by colored asterisks. Data shown is the average of 3 or greater independent experiments. Error bars show the standard deviation. Significance levels are as follows: *, *P* < 0.05; **, *P* < 0.01.

In previous studies, selection for resistance to PF74 resulted in two distinct CA variants, CA 4-mut and CA 5-mut, which share a single CA Q67H mutation (6, 26). These combinational CA mutants were not observed during selection for LCV-resistant HIV-1; however, several single amino acid substitutions that are components of either CA 4-mut or CA 5-mut were acquired (8). We were curious how PF74-resistant HIV-1 mutants would fare in the presence of LCV and how inositol phosphate depletion would affect the LCV resistance profile. To this end, WT_Vector_, IPMK KO_Vector_, and IPMK KO_IPMK-Flag_ CEM cells were used as target cells for infection with HIV-1_CA 4-mut_ or HIV-1_CA 5-mut_ in the presence of increasing doses of LCV. Infection in the absence of the drug was initially characterized. As previously shown, complementation of IPMK partially rescued the >2-fold defect in HIV-1_CA WT_ infection observed in IPMK KO_Vector_ cells (Fig. 3A, black bars). Independent of cell line, infection by the HIV-1_CA 4-mut_ virus was ∼60% that of HIV-1_CA WT_ (Fig. 3B, light green dashed line). However, the HIV-1_CA 5-mut_ virus demonstrated a small but significant increase in the infection of IPMK KO_Vector_ target cells compared to the infections of both the WT_Vector_ and IPMK KO_IPMK-Flag_ cells (Fig. 3B, green dashed line), suggesting that the combination of mutations in the CA 5-mut capsid may be beneficial to infection of IP6-depleted target cells.

We next assessed the susceptibility of HIV-1_CA 4-mut_ and HIV-1_CA 5-mut_ to inhibition by LCV. at a concentration of 1.56 nM LCV strongly inhibited HIV-1_CA WT_ infection of all cell lines tested with IP5/IP6 codepletion completely inhibiting infection (Fig. 3C). Comparatively, in all cell lines tested, both CA mutant HIV-1 viruses infected a large proportion of cells in the presence of 1.56 nM LCV, with HIV-1_CA 4-mut_ being ∼50% less infectious and HIV-1_CA 5mut_ being unaffected by the drug (Fig. 3C). In WT and complemented cell lines, the dose-response curves for the CA 4-mut and CA 5-mut viruses were substantially altered from that of HIV-1_CA WT_, displaying a pronounced plateau phase and a gradual slope of inhibition at the most efficient LCV concentrations (Fig. 3C and Table 6). For HIV-1_CA 4-mut_, the IC_50_ in WT_Vector_ cells was ∼50-fold greater and the slope at high LCV concentrations changed from 1.9 for HIV-1_CA WT_ to 0.88 for HIV-1_CA 4-mut_ virus (Table 6). HIV-1_CA 5-mut_ resistance to LCV was even more pronounced in WT_Vector_ cells, with an ∼900-fold increase in IC_50_ and the slope changed to ∼1.2 at high LCV concentrations (Table 6). These decreases in LCV potency and antiviral activity were maintained for both CA mutants in IPMK-complemented cells. IPMK KO had little effect on the IC_50_ of LCV for either CA mutant (Table 6). However, IP5 and IP6 depletion altered the antiviral efficiency of LCV against the CA mutant viruses, changing the slope by 3-fold for each mutant virus at high LCV concentrations (Fig. 3C; Table 6, m′). These results further confirm that IP6 affects HIV-1 infection in target cells in the presence of LCV. Furthermore, our data show that IP6 depletion can help promote inhibition of LCV-resistant HIV-1.

**TABLE 6:** Hill equation parameters and fit for the effect of IP5/IP6 depletion on LCV inhibition of PF74-resistant HIV-1 in CEM cells

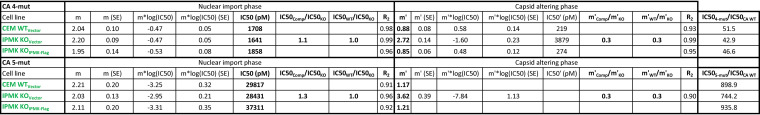

### IP6 prevents HIV-1 capsid destabilization by PF74.

HIV-1 infection was more effectively inhibited upon IP6 depletion at concentrations of LCV or PF74 that inhibit RTN. At these concentrations, the inhibitors can alter capsid stability (7, 9, 27). We therefore hypothesized that IP6 might counteract the capsid-altering activities of PF74 and LCV. To test this, we performed *in vitro* uncoating assays using purified HIV-1 cores under conditions that support efficient RTN *in vitro* (16). Purified HIV-1 cores were incubated in reactions containing deoxyribonucleotide triphosphates (dNTPs) in the presence of various concentrations of IP6, PF74, and LCV. Reactions were diluted in cold buffer and the cores pelleted by ultracentrifugation. The levels of CA or reverse transcriptase (RT) in the pellets, and CA in the supernatant, were assayed to determine the amount of each protein released from the cores during the reaction.

**FIG 4:**
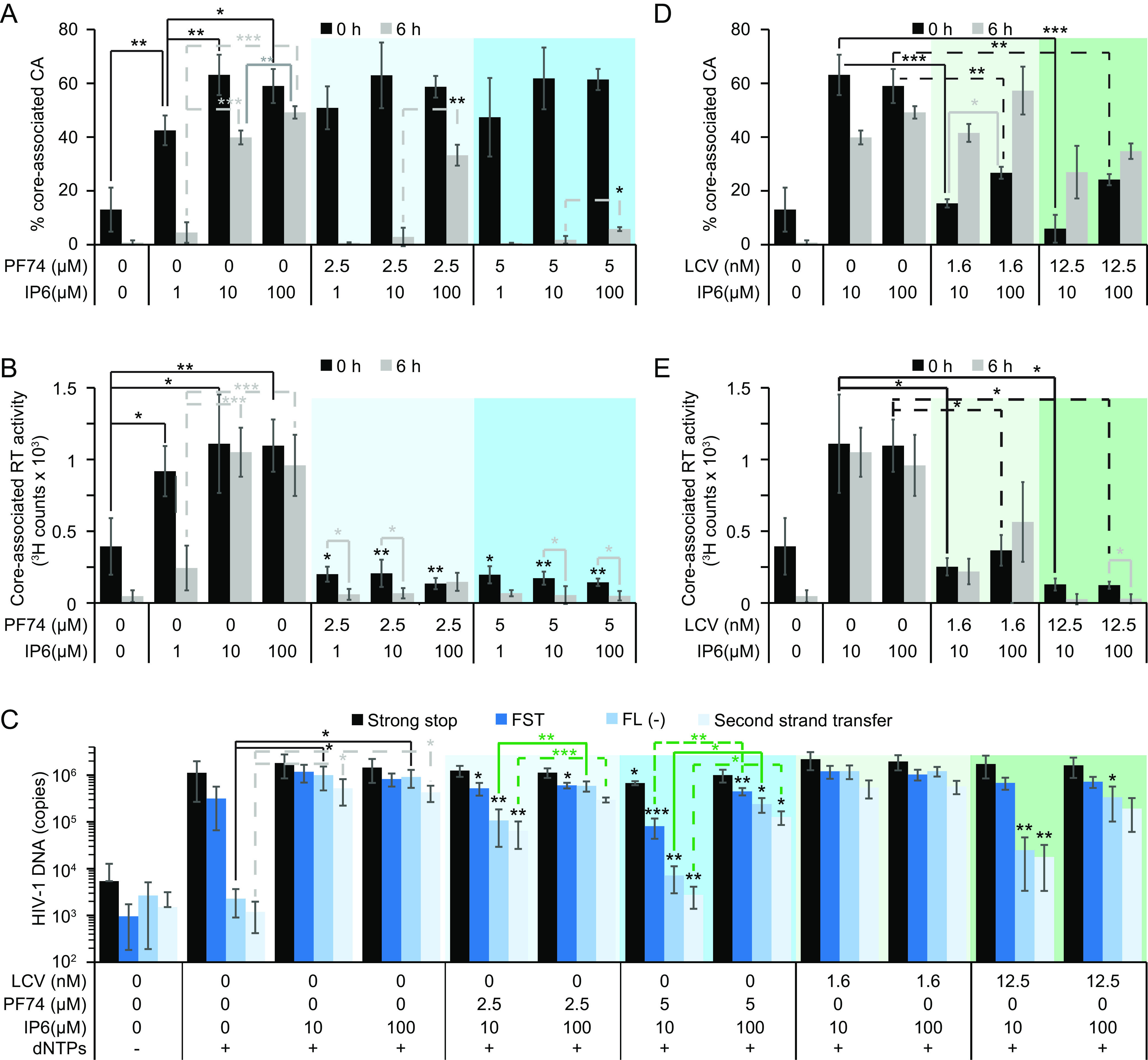
IP6 abrogates the PF74 and LCV effects on premature HIV-1 core uncoating *in vitro.* (A, B, D, and E) Uncoating of purified HIV-1 cores measured by HIV-1 p24 ELISA (A and D) or exogenous RTN of pelleted cores (B and E). Controls lacking inhibitors are the same in panels A and D (and B and E). Brackets show statistical significance in the indicated comparison. Statistical comparisons lacking brackets compare the indicated condition to the same condition lacking CA inhibitor. (D and E) Statistical comparisons for cores lacking LCV are not shown, but *P* values are the same as those shown in panels A and B, respectively. (C) Quantification of ERT by qPCR. Significant comparisons in the absence of PF74 or LCV are shown using brackets. For comparisons lacking brackets, black asterisks indicate significance differences in the indicated RTN product between the marked bar and the corresponding vehicle-treated bar containing the same amount of IP6. (A, B, and E) Each bar consists of the average of 3 independent experiments with corresponding error bars for standard deviation. Panels D and E represent 2 independent experiments along with error calculated using standard deviation. Significance levels are as follows: *, *P* < 0.05; **, *P* < 0.01; ***, *P* < 0.001.

In reactions lacking IP6, a substantial fraction of both CA and RT were quickly shed from cores (Fig. 4A and B, 0 h). Upon incubation at 37°C for 6 h, most of the core-associated CA and RT decreased to background levels, consistent with nearly complete uncoating. Addition of IP6 to the reactions reduced the dissociation of both CA and RT from the core, with 10 μM IP6 resulting in retention of substantial quantities of both CA and RT during the incubation (Fig. 4A and B). We observed little CA or RT loss over the time course in reactions containing 10 and 100 μM IP6. Thus, consistent with previous results (12, 16), we conclude that IP6 stabilizes HIV-1 cores in ERT reactions, preserving the association of RT for at least 6 h.

We next assessed the effect of PF74 and IP6 on HIV-1 uncoating. In reactions containing IP6 and PF74, CA remained core associated at the start of the reaction. However, during incubation, PF74 promoted the release of both CA and RT relative to control reactions lacking the compound (Fig. 4A and B). In reactions containing 100 μM IP6, the uncoating effect of PF74 was reduced. This was most apparent in reactions containing 2.5 μM PF74 in which the addition of 100 μM IP6 markedly reduced CA loss from the core (Fig. 4A, compare 100 to 10 μM IP6 ± 2.5 μM PF74). Independent of IP6 concentration and in the presence of PF74, pelletable RT decreased immediately upon addition of cores to the uncoating reaction (Fig. 4B). Increasing the IP6 concentration from 10 to 100 μM prevented the loss of the remaining RT from the core when 2.5 μM PF74 was included but not 5 μM PF74 (Fig. 4B). Our results indicate that PF74 destabilizes the HIV-1 capsid, and this effect is reduced by IP6.

We also tested the effects of varying IP6 and PF74 concentrations on ERT. As previously reported (16, 17), reactions lacking IP6 failed to produce FLM and second strand transfer (SST) late RTN products but were proficient for strong stop (SS) and FST RTN synthesis (Fig. 4C). The addition of IP6 robustly stimulated synthesis of late products (Fig. 4C). PF74 inclusion in reactions containing 10 μM IP6 inhibited late RTN product synthesis and could be fully or partially rescued by adding 100 μM IP6 in the reaction (Fig. 4C). Thus, IP6 stabilizes the HIV-1 capsid *in vitro* to permit RTN even in the presence of PF74.

### The HIV-1 capsid is stabilized by IP6 to antagonize LCV *in vitro*.

In parallel, we also determined the effects of the more potent inhibitor LCV on the uncoating of purified HIV-1 cores *in vitro*. Unlike PF74, LCV elicited CA loss from the core immediately upon addition to the reaction (Fig. 4D). At both LCV concentrations tested, increasing the IP6 concentration from 10 to 100 μM decreased the amount of CA shedding that occurred without incubation (Fig. 4D). Surprisingly, compared to no incubation, incubation at 37°C increased the amount of core-associated CA in reactions containing LCV, suggesting the possibility of CA reassembly under these conditions (Fig. 4D). Thus, LCV elicited CA uncoating immediately upon addition to the reaction, an effect that was reduced by 100 μM IP6.

Like CA, RT was released from the HIV-1 cores immediately upon LCV addition regardless of the amount of IP6 included. However, increasing the amount of IP6 from 10 to 100 μM in the presence of 1.56 nM LCV slightly increased the amount of pelletable RT with and without incubation (Fig. 4E). Incubation of uncoating reactions containing 12.5 nM LCV caused nearly complete dissociation of RT upon incubation regardless of the IP6 concentration, suggesting that at this LCV concentration the capsid becomes more porous (Fig. 4E). We conclude that LCV inhibits RTN by destabilizing the core resulting in RT leakage and that IP6 can partially counteract this activity *in vitro* by stabilizing the capsid.

To understand the effect of LCV on ERT, we initially tested a wide range of LCV concentrations in ERT reactions that inhibit RTN in cells (Fig. 2C and D). Unexpectedly, 0.78 to 6.25 nM LCV had no effect on ERT in reactions containing IP6 (Fig. 4C). While HIV-1 infection was nearly completely inhibited by 3 nM LCV, 12.5 nM LCV substantially inhibited synthesis of late RTN products in ERT relative to reactions containing 10 μM IP6 and lacking the drug (Fig. 4C). However, increasing the amount of IP6 from 10 to 100 μM reduced the inhibition by 12.5 nM LCV, suggesting that IP6 partially counteracted the destabilizing effects of LCV on the HIV-1 capsid. Our *in vitro* data using LCV and purified HIV-1 cores demonstrate that LCV destabilizes the HIV-1 capsid and that physiological concentrations of IP6 can reverse the effect, thereby reducing inhibition of ERT.

## DISCUSSION

**FIG 5:**
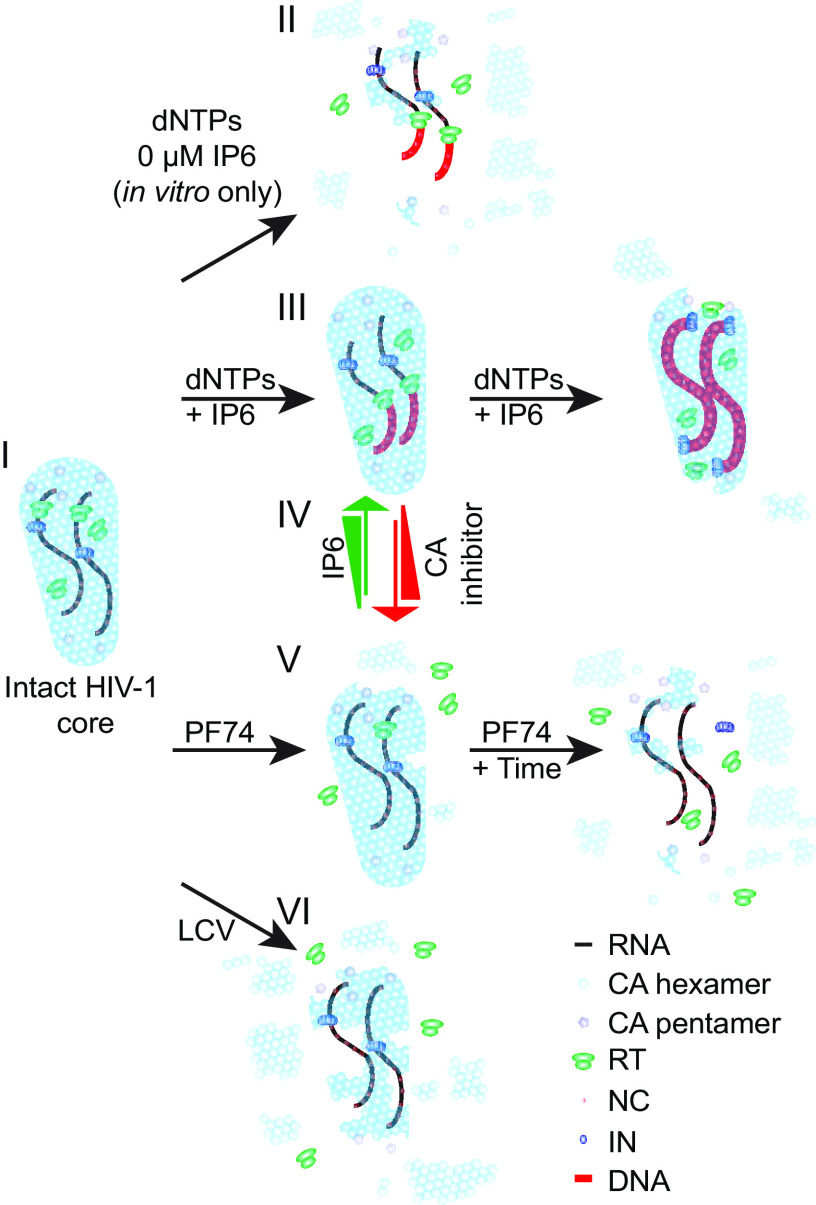
The interplay of IP6 and CA inhibitors. Model of HIV-1 uncoating in the presence of IP6, CA inhibitors, and/or dNTPs. (I) An intact HIV-1 core is released into the cell or added to a reaction. (II) When no IP6 is present in ERT reactions, the core quickly uncoats. The core is proficient to generate early RTN products *in vitro*. (III) HIV-1 cores in the cell and *in vitro* are stabilized by IP6 and polymerize full-length HIV-1 cDNA. (IV) The balance of IP6 and CA inhibitor concentrations alters the flux between HIV-1 being RTN proficient with a stable capsid (promoted by IP6) and inhibiting HIV-1 RTN via capsid destabilization (CA inhibitor). (V) PF74 addition to the core immediately elicits RT loss from the core. Upon incubation the core disassembles. (VI) LCV binding to the core causes abrupt uncoating.

The relevance of HIV-1 CA binding to IP6 in cells has been unclear since the initial observation that IPPK or IPMK KO in HEK293T cells had no effect on target cell infection (19). IP6 stimulates assembly of CA into mature-like particles *in vitro* (28); however, only circumstantial evidence of a cellular role for the CA/IP6 interaction has been reported (18). In the current study, we observed that IP6 depletion in the presence of CA inhibitors increases the antiviral effect of LCV and PF74 concentrations that inhibit RTN by destabilizing the capsid (Fig. 1, 2, and 4; Tables 1 to 4). The enhanced antiviral activity was associated with more potent inhibition of RTN in IP6-depleted target cells. Further, the same capsid-destabilizing CA inhibitor concentrations that display increased antiviral activity in IP6-depleted cells can be partially counteracted by increasing the IP6 concentration in both uncoating and ERT reactions *in vitro* (Fig. 4). In these uncoating reactions, increasing the amount of IP6 in the presence of CA inhibitors partially prevented uncoating, thereby stabilizing the capsid to promote ERT (Fig. 4 and Fig. 5, IV).

Taken together, these results support the conclusion that IP6 in target cells limits the antiviral activity of PF74 and LCV by stabilizing the viral capsid, with each small molecule exerting a differential effect on the capsid at their respective, distinct binding sites (Fig. 5) (3, 7, 8, 29). While the consequences of capsid stabilization by IP6 are quite subtle in the absence of inhibitors, they become more apparent in the presence of capsid-destabilizing compounds (Fig. 1 and 2). Interestingly, the antiviral activity of another CA-targeting inhibitor, BI-2, was unaffected by IP6 depletion (Fig. 2E), consistent with the inability of this compound to destabilize the native HIV-1 capsid in cells and inhibit RTN (25). Thus, further capsid destabilization is required in IP6-depleted cells to reveal a defect in target cell infection. Our data demonstrate that IP6 is directly affecting the HIV-1 capsid at RTN during uncoating, and the function of IP6 binding to CA in cells is to stabilize the core (Fig. 5).

### HIV-1 capsid-stabilizing cofactors.

The requirement for CA inhibitors to reveal the cryptic capsid-stabilizing role of IP6 in target cells hints that, in target cells, other CA interactions, i.e., those at the PF74/LCV binding site, are sufficient to stabilize the capsid in cells (2). Alternatively, in IPPK and IPMK KO cells, residual IP6 may be sufficient to stabilize the HIV-1 capsid. Consistent with the first hypothesis, SEC24C was identified to bind to the PF74/LCV binding site in CA (2). The SEC24C/CA interaction stabilizes the HIV-1 capsid *in vitro* and in target cells, and SEC24C KO or knockdown leads to a marginal defect in HIV-1 infection, a phenotype comparable to IP5 and IP6 depletion (Fig. 1A and B) (2). Thus, it seems plausible that SEC24C is the capsid-stabilizing factor blocked from binding to CA at capsid destabilizing concentrations of CA inhibitors. Nonetheless, the effect(s) of NUP153 and CPSF6 binding to CA on capsid stability remain ambiguous; thus, it remains to be determined which host factor(s), if any, regulate HIV-1 capsid stability during infection.

Residual inositol phosphates in our KO cell lines may be sufficient to stabilize the capsid, thereby providing enough IP5/IP6 for capsid stabilization during RTN, resulting in successful HIV-1 infection (Fig. 1A and B). Consistently, IP5 accumulates in IPPK KO cells (18, 19), and low amounts of IP6 remain in IPMK KO cells (18, 19). Cytotoxic ectopic expression of multiple inositol-polyphosphate phosphatase 1 (MINPP1) in IPPK KO HEK293FT cells depleted remaining IP5/IP6 and suggested that removing residual IP5/IP6 from IPPK KO cells exacerbates the HIV-1 release defect in IPPK KO cells (30). This latter conclusion is limited by the toxic effects of MINPP1 overexpression in IPPK KO cells along with the low relevance of the SV40 large T antigen overexpressing HEK293FT cell line used (30). Other cell metabolites might also bind to Arg18 to stabilize the HIV-1 capsid in target cells upon IP5 and/or IP6 depletion; however, this remains to be tested. CA inhibitors were less efficient in IPPK KO cells than IPMK KO cells, regardless of cell line or clone tested (Fig. 1 and 2). Both IP5 and IP6 can stimulate/stabilize CA assembly *in vitro* (28); thus, different IP5 and IP6 concentrations could explain the differences observed between IPPK KO and IPMK KO cell lines (18, 19). Based on our cellular data, we suggest that IP5, like IP6, can stabilize the HIV-1 capsid in cells similar to its ability to replace IP6 in stimulation of HIV-1 release from IPPK KO HEK293T cells (19).

### LCV compared to PF74.

Both PF74 and LCV exhibited biphasic HIV-1 inhibition curves in T cells with high concentrations of each inhibitor inhibiting RTN (Fig. 1 and 2). Previous *in vitro* studies demonstrated that PF74 concentrations that inhibit RTN also destabilize the viral capsid (Fig. 5) (9, 16, 27). Using cellular assays, we identified PF74 concentrations whose destabilization was modulated by IP6 concentration (Fig. 1, Fig. 4A and B, and Fig. 5, IV). However, a previous mechanistic study of the antiviral effects of LCV against HIV-1 concluded that LCV blocks RTN in cells at concentrations that stabilize the HIV-1 capsid (7). At odds with this interpretation, HIV-1 capsids containing stabilizing mutations are proficient for RTN but fail at nuclear entry (11). The assays and LCV concentrations used to assess capsid stability differ between reference (7) and our study, which might explain why we obtained differing conclusions. Further, it remains possible that LCV functions differently in a cellular context. However, we present evidence of CA and RT release from HIV-1 cores *in vitro* in the presence of LCV (Fig. 4D and E). Collectively, our results support a model wherein both PF74 and LCV inhibit RTN by destabilizing the HIV-1 capsid, resulting in premature RT leakage (Fig. 5, V and VI).

Although both PF74 and LCV elicited immediate loss of RT from cores *in vitro* (Fig. 4A, B, D, and E), these compounds appear to differentially promote uncoating in our assays. Both compounds released RT from the core upon addition (Fig. 4B and E). Yet, whereas LCV elicited CA uncoating upon addition to the reaction, PF74 required incubation at 37°C (Fig. 4A, B, D, and E; Fig. 5, V and VI). LCV promoted what may be reassembly of pelletable, RT-free CA in uncoating reactions (Fig. 4D and E), perhaps explaining why, in the initial characterization of LCV, the authors concluded that the compound stabilizes the capsid (7).

The destabilizing activities of each CA inhibitor were partially reversed by increasing the amount of IP6 in uncoating reactions, suggesting that further capsid stabilization by IP6 can antagonize PF74 and LCV (Fig. 5, IV). Our study of PF74-resistant HIV-1 mutants reaffirmed this conclusion, and the effect of IP6 on LCV antiviral activity against each PF74-resistant mutant was exacerbated compared to that of HIV-1_CA WT_ (Fig. 3 and Table 6). Our results argue that, although CA 4-mut and CA 5-mut viruses are up to 900-fold more resistant to LCV than WT HIV-1 and are nearly fully infectious (Fig. 3 and Table 6), these viruses remain dependent on IP6 for proper capsid stability as indicated by the CA mutant’s susceptibility to IP5 and IP6 depletion upon dosing with CA inhibitors. We suggest that perturbing the IP6 binding site in CA with small molecules might be a robust strategy for targeted next-generation antiretroviral therapies.

## MATERIALS AND METHODS

### Cells and viruses.

All CEM and MT-4 cell lines were as previously described (18) and maintained in complete RPMI (RPMI 1640 containing 10% fetal bovine serum [FBS] and 12.5 mg/ml Blasticidin S [InvivoGen]). HEK293T cells were maintained in Dulbecco modified Eagle medium (DMEM) containing 10% FBS. HIV-1-GFP_CA WT VSVg_, HIV-1-GFP_CA 4-mut VSVg_, HIV-1-GFP_CA 5-mut VSVg_, and VSVg-pseudotyped HIV-1_NL4-3_Δenv were produced by polyethylenimine transfection (Polysciences, Inc.) of HEK293T as previously described (9, 18, 26). Viruses were titered by p24 enzyme-linked immunosorbent assay (ELISA) and flow cytometry (18).

### HIV-1 infection.

For all HIV-1 infections, data averages with standard deviations for 3 or more independent experiments are shown. In all experiments, CEM and MT-4 T cell lines were used as target cells for HIV-1 infection. To assay target cell infection, 200,000 CEM or MT-4 cells were plated in the presence of the indicated amounts of PF74 (Pfizer), LCV (MedChemExpress), EFV (NIH AIDS reagent program), raltegravir (NIH AIDS reagent program), BI-2 (Vanderbilt University Molecular Design and Chemical Synthesis Center), or an equivalent amount of vehicle (dimethyl sulfoxide [DMSO], Sigma). Cells were subsequently infected with 3 ng p24 of HIV-1-GFP_VSVg_. At 2 days postinfection, the cells were fixed overnight at 4°C in 2% (weight per volume) paraformaldehyde (Sigma). Infection was measured using a FACS Canto II flow cytometer (BD), and data were analyzed using the FlowCore package in R (31). To compare the data between the various inhibitor-treated cell lines, data were internally normalized as the percentage of vehicle (% infected_drug-treated_/% infected_vehicle-treated_).

To quantify HIV-1 RTN in cells, 1,000,000 CEM cells were plated into complete RPMI media containing the indicated amount of each drug and infected with 40 ng p24 of DNase I-treated VSVg pseudotyped HIV-1_NL4-3_Δenv. At 2 h postinfection, cells were pelleted at 700 × *g* for 5 min, and the supernatant was removed. After washing the cells one time in phosphate-buffered saline (PBS), cells were resuspended in complete RPMI-containing vehicle (DMSO) or the indicated drug. At 8 h, the cells were harvested, and DNA was extracted using a silica gel column (Epoch Life Sciences) as described in reference (32). Three microliters out of 60 μl of the extracted ERT reaction cDNA were used per qPCR. HIV-1 RTN qPCR in cells was performed using 100 ng of genomic DNA as input into the qPCR. The amount each RTN product was quantified by absolute quantification on an Mx3000P multiplex quantitative PCR system (Stratagene) using Maxima SYBR green/ROX qPCR master mix (Thermo Scientific) and the primers described in Table 7.

**TABLE 7:** Summary of oligonucleotides used in this study

Name	Purpose	Sequence (5′ to 3′)
NL4-3 FST-F1	qPCR of FST HIV-1 RTN in cells	GAG CCC TCA GAT GCT ACA TAT
SS-R4	qPCR of FST HIV-1 RTN in cells and by ERT	CCA CAC TGA CTA AAA GGG TCT GAG
1512F	qPCR of FLM HIV-1 RTN in cells and by ERT	ACC ATG CTA AAC ACA GTG GG
1563R	qPCR of FLM HIV-1 RTN in cells and by ERT	AGC TTC CTC ATT GAT GGT CTC
hRU5-F2	qPCR of SS HIV-1 ERT products	GCC TCA ATA AAG CTT GCC TTG A
hRU5-R	qPCR of SS HIV-1 ERT products	TGA CTA AAA GGG TCT GAG GGA TCT
R9 FST-F1	qPCR of FST HIV-1 ERT products	GAG CCC TCA GAT CCT GCA TAT
MH531	qPCR of SST HIV-1 ERT products	TGT GTG CCC GTC TGT TGT GT
MH532	qPCR of SST HIV-1 ERT products	GAG TCC TGC GTC GAG AGA GC

### HIV-1 core purification, uncoating, and ERT reactions.

HIV-1_R9_ cores were purified from infected MT-4 cells as previously described (16, 33). Five or 10 ng p24 of purified HIV-1 cores were used in ERT or uncoating assays, respectively, as described in reference 16. ERT products were quantified by qPCR as described in the previous section. HIV-1 uncoating was measured by p24 ELISA and exogenous-RT assays as previously described (16). Reactions lacking either CA inhibitor contained DMSO. A minimum of 3 independent experiments were performed for HIV-1 uncoating and ERT assays. Data in Fig. 4A to C represent the average of 3 or more experiments with error bars denoting standard deviation. HIV-1 core uncoating in the presence of LCV was performed using 2 separate HIV-1 core purifications in 4 independent experiments. The average of 2 independent experiments with standard deviation from one of the core preparations is shown in Fig. 4D and E.

### Statistics.

Prior to pairwise *t* tests, a one-way analysis of variance (ANOVA) was applied to data using the Microsoft Excel Data Analysis package. To compare two experimental conditions, Welch’s test was performed in R. Where applicable, *P* values were corrected for multiple comparisons using the Hommel method in R. Significance levels are as follows: *, *P* < 0.05; **, *P* < 0.01; ***, *P* < 0.001; ****, *P* < 0.0001.

### Calculation of dose-response curve parameters.

To fit dose-response curves to the Hill equation, the *y* axis data (% of vehicle [*f_u_*]) was transformed to (1 − *f_u_*)/(*f_u_*). Both *x* (drug concentration [D]) and *y* axis data were then log_10_ transformed. Log_10_ transformed data were plotted in Microsoft Excel, and each linear phase containing 3 or more drug concentrations (9 or more data points per line) was used for a linear regression using the Regression function in the Microsoft Excel Data Analysis package. The linear regression was used to fit the linearized monophasic Hill equation as follows: log_10_[(1 − *f_u_*)/(*f_u_*)] = m*log(D) − m*log_10_(IC_50_). The slope (m) corresponds to the Hill coefficient and dictates the steepness of inhibition. The IC_50_ was calculated by IC_50_ = 10^−(^*^y^*^-intercept)/m^. For biphasic curves (i.e., PF74 and LCV), each phase of the dose-response curve was used for a linear regression with the initial phase yielding the IC_50_. The second phase of the biphasic dose-response curve yields information about the final slope of inhibition m′ and corresponds to the relationship between HIV-1 infection and the drug concentration (34). Results of fitting the transformed dose-response data to a monophasic Hill equation are shown in Tables 1 to 6.
